# Nasal Arteriovenous Malformation Presenting With Sebaceous Hyperplasia on Superficial Biopsy: A Case of Clinicopathological Discordance

**DOI:** 10.1002/ccr3.72895

**Published:** 2026-06-07

**Authors:** Qazi Syed Irfanullah Shah, Xuefeng Wan, Palida Abliz, Akebaier Sulaiman

**Affiliations:** ^1^ The First Affiliated Hospital of Xinjiang Medical University Urumqi China

**Keywords:** arteriovenous malformation, clinicopathological discordance, nasal AVM, sebaceous gland hyperplasia, vascular malformation

## Abstract

Persistent facial lesions with benign superficial histopathology should prompt suspicion of deeper vascular anomalies. Clinicopathological discordance warrants early vascular imaging, as superficial biopsy may fail to detect underlying arteriovenous malformations, leading to delayed diagnosis and inappropriate management.

## 1 Introduction

Arteriovenous malformations (AVMs) are rare congenital vascular anomalies that arise from abnormal embryologic development of the vascular system and are characterized by direct connections between arteries and veins without an intervening capillary bed [1]. These lesions are typically high‐flow in nature and may progressively enlarge over time, leading to functional impairment, cosmetic deformity, or bleeding complications [1, 2].

Although AVMs can involve various organs, lesions affecting the head and neck region are relatively uncommon, with nasal arteriovenous malformations representing a particularly rare subset [2, 3]. The clinical presentation of nasal AVMs is highly variable and may include epistaxis, nasal obstruction, facial swelling, or cutaneous erythema [4]. However, atypical presentations without classical vascular signs have been reported and may closely mimic benign dermatological or inflammatory conditions, resulting in delayed or incorrect diagnosis [3, 5].

Sebaceous gland hyperplasia is a common benign adnexal condition of the face and is rarely associated with underlying vascular anomalies [6]. When nasal AVMs present with predominant cutaneous or adnexal features, clinical suspicion for a vascular lesion may be low, particularly in the absence of pulsation or bleeding [3, 5]. Such atypical presentations pose a diagnostic challenge and highlight the limitations of relying solely on clinical examination or superficial histopathology.

We report a rare case of nasal arteriovenous malformation presenting primarily as sebaceous gland hyperplasia, emphasizing the importance of clinical radiological correlation and awareness of unusual dermatological manifestations of vascular anomalies.

## 2 Case History/Examination

A 24‐year‐old woman presented to the dermatology clinic with a persistent unilateral nasal lesion that had been gradually enlarging over the past five years. The lesion initially appeared as a small erythematous papule, approximately the size of a rice grain, located on the nasal bridge. Over time, it slowly expanded across the nasal dorsum while maintaining a stable color and texture. The patient reported no associated pain, bleeding, ulceration, pulsation, or nasal obstruction. There was no history of recurrent epistaxis, facial trauma, surgery, or systemic illness.

**FIGURE 1:**
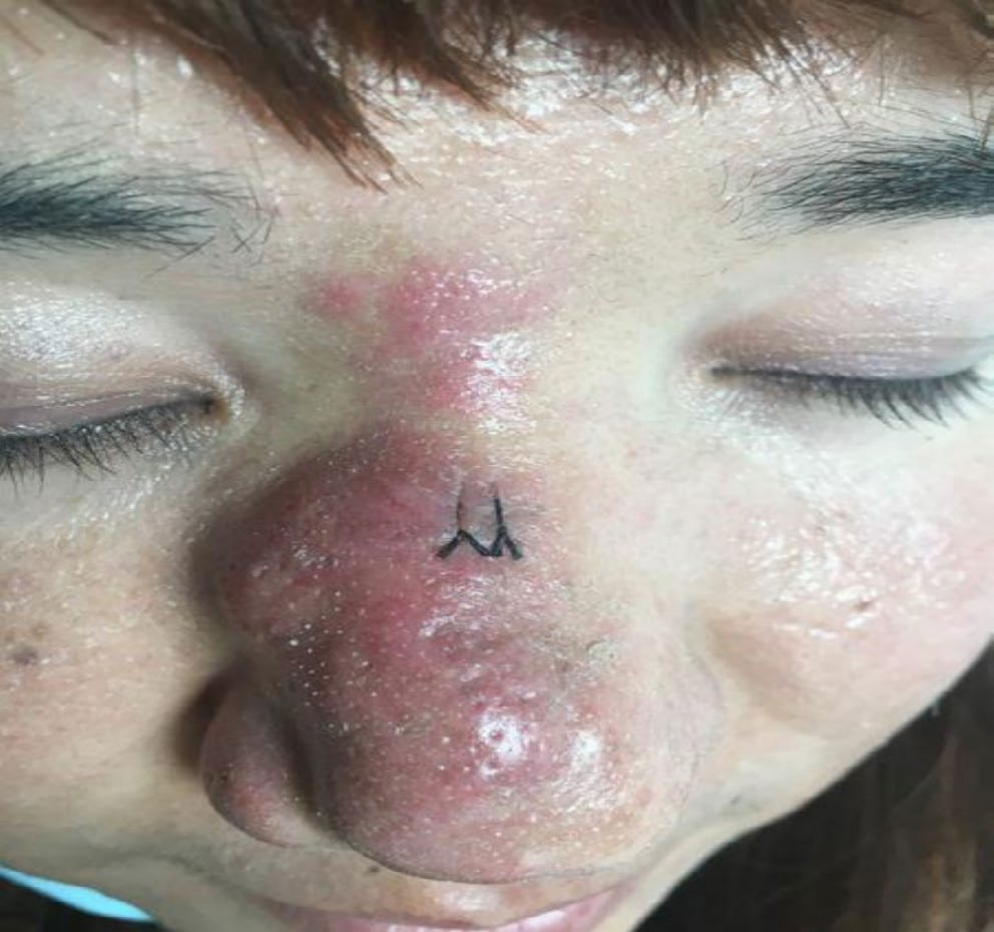
Clinical presentation of the nasal lesion. Localized erythematous plaque involving the nasal dorsum, demonstrating mild surface irregularity without ulceration, crusting, or bleeding.

On physical examination, a localized erythematous plaque with mild surface irregularity was observed on the nasal dorsum. The lesion was nontender, noncompressible, and nonpulsatile, with no audible bruit. The surrounding skin appeared normal, and there was no regional lymphadenopathy. Systemic examination was unremarkable (Figure 1).

## 3 Differential Diagnosis, Investigations and Treatment

Based on the clinical findings, the initial differential diagnoses included granulomatous dermatosis, sebaceous gland hyperplasia, rosacea, adnexal tumor, and vascular anomaly. A punch biopsy (approximately 3–4 mm in diameter) was obtained from the superficial portion of the lesion over the nasal dorsum to establish a definitive diagnosis.

**FIGURE 2:**
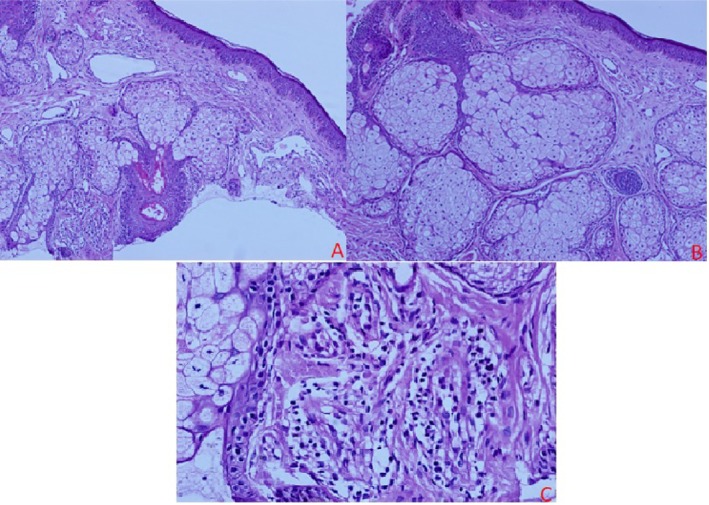
Histopathological findings of the nasal lesion. (A) Low‐power photomicrograph showing prominent sebaceous gland hyperplasia within the dermis. (B) Intermediate magnification demonstrating enlarged and increased sebaceous lobules arranged in clusters. (C) High‐power view confirming mature sebaceous glands without significant inflammatory infiltrate, cytologic atypia, or malignant features (hematoxylin and eosin stain).

Histopathological examination demonstrated prominent sebaceous gland hyperplasia without evidence of malignancy, granulomatous inflammation, or significant vascular proliferation (Figure 2). However, the biopsy was limited to the superficial dermis and did not include deeper tissue layers, which likely contributed to the absence of identifiable vascular components. Given that arteriovenous malformations are typically located in the deeper dermis and subcutaneous tissue, the superficial sampling may have resulted in a misleading benign histopathological finding.

This clinicopathological discordance raised suspicion for an underlying deeper vascular pathology not adequately sampled by superficial biopsy. Given the discrepancy between the long‐standing clinical course and the benign histological findings, further radiological evaluation was pursued.

**FIGURE 3:**
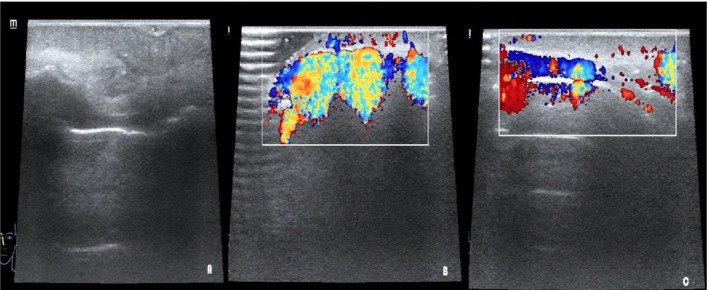
Doppler ultrasonographic evaluation of the nasal lesion. (A) Gray‐scale ultrasound image showing altered soft‐tissue echotexture in the nasal region. (B) Color Doppler image demonstrating marked internal vascularity with multiple tortuous vascular channels. (C) Color Doppler view showing high‐flow vascular channels consistent with an arteriovenous malformation.

Given the persistent progression of the lesion and discordance between clinical findings and histopathology, further imaging was undertaken. Color Doppler ultrasonography revealed increased internal vascularity with high‐flow signals suggestive of a vascular malformation. Contrast‐enhanced computed tomography confirmed abnormal arteriovenous connections consistent with a nasal arteriovenous malformation (Figure 3).

**FIGURE 4:**
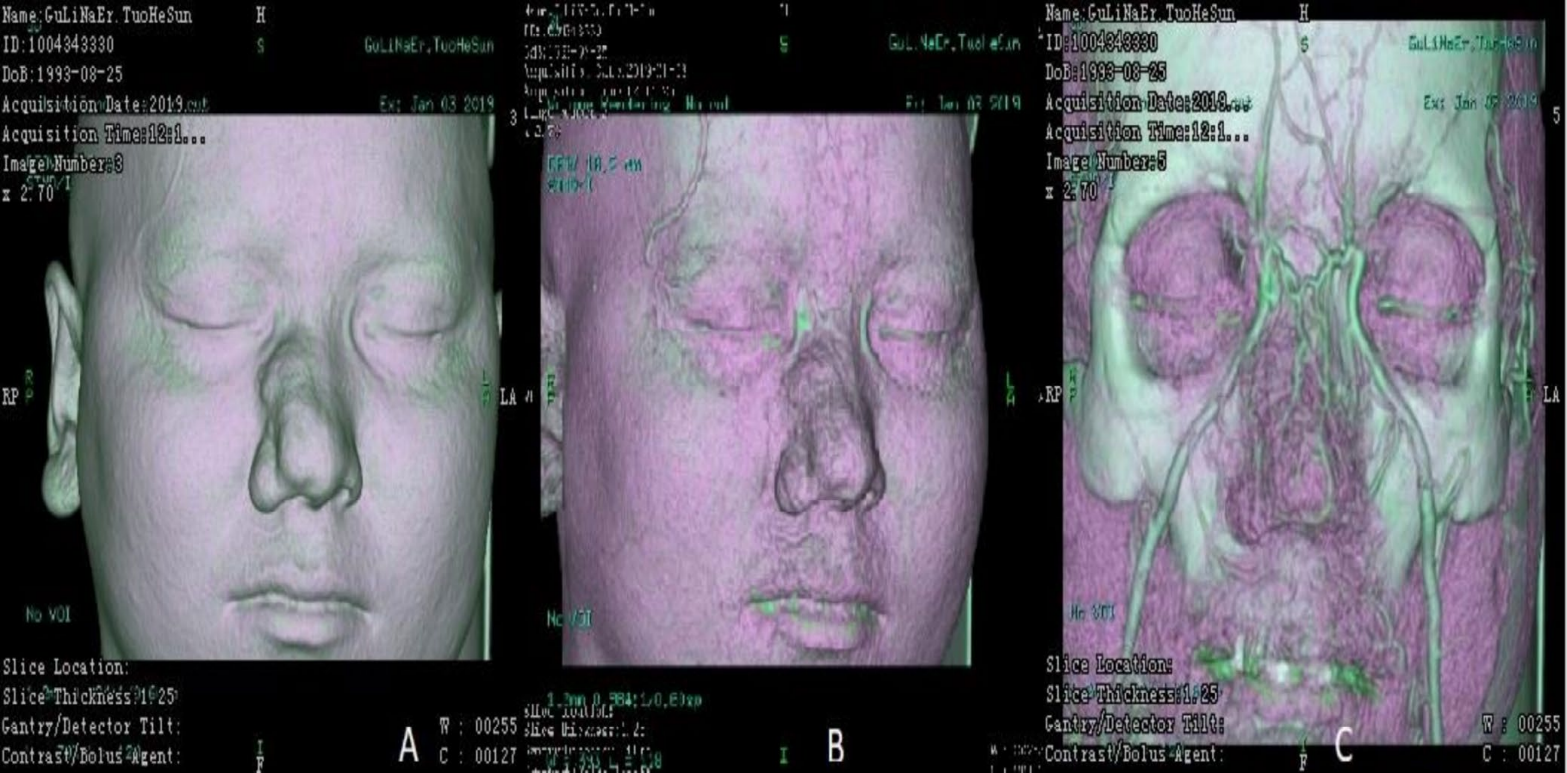
Contrast‐enhanced computed tomography with three‐dimensional vascular reconstruction of the nasal region. (A) Three‐dimensional surface reconstruction demonstrating the anatomical contour of the nasal region. (B) Vascular reconstruction revealing abnormal tortuous vascular channels in the nasal dorsum. (C) Enhanced vascular visualization showing prominent feeding vessels and abnormal arteriovenous connections consistent with an arteriovenous malformation.

Subsequent contrast‐enhanced computed tomography with three‐dimensional angiographic reconstruction demonstrated abnormal high‐flow vascular channels and feeding vessels in the nasal region, confirming the diagnosis of a nasal arteriovenous malformation (Figure 4). The vascular channels were noted to extend beyond the superficial dermis into the deeper subcutaneous tissue, supporting the presence of a high‐flow vascular malformation and explaining the absence of vascular features on superficial biopsy.

The patient initially underwent intralesional sclerotherapy using lauromacrogol; however, no significant clinical improvement was observed. Subsequently, surgical ligation of the feeding vessel was performed, resulting in marked regression of the lesion.

## 4 Conclusion and Results (Outcome and Follow‐Up)

Following surgical ligation of the feeding vessel, the lesion showed marked regression. No recurrence or progression was observed during a six‐month follow‐up period. The outcome highlights the importance of accurate diagnosis and appropriate management in high‐flow vascular malformations.

## 5 Discussion

This case is notable for significant clinicopathological discordance, which posed a diagnostic challenge. Arteriovenous malformations are rare high‐flow vascular anomalies characterized by direct arteriovenous shunts without an intervening capillary bed [1]. Lesions involving the head and neck region, particularly the nasal area, are uncommon [2].

Clinically, nasal AVMs may present with epistaxis, nasal obstruction, facial swelling, or cutaneous erythema; however, atypical presentations lacking classical vascular features can lead to delayed diagnosis [4, 5]. In the present case, the lesion showed slow progression over several years without pain or bleeding, mimicking a benign dermatological condition.

Histopathological evaluation of persistent lesions may be misleading when superficial biopsy fails to capture deeper vascular components. In this case, biopsy revealed sebaceous gland hyperplasia without vascular proliferation, reflecting the limitation of superficial sampling in detecting underlying AVMs [6, 7].

Imaging plays a crucial role in the diagnosis of AVMs. Doppler ultrasonography demonstrates high‐flow vascular signals, while contrast‐enhanced computed tomography provides detailed assessment of lesion extent and vascular anatomy, particularly when clinicopathological findings are inconclusive [8, 9].

Management of AVMs requires a multidisciplinary approach. In this case, sclerotherapy with lauromacrogol was ineffective, likely due to the high‐flow nature of the lesion, whereas surgical ligation of the feeding vessel resulted in clinical improvement [4, 5].

This case broadens the clinical spectrum of nasal arteriovenous malformations and highlights that atypical or treatment‐resistant sebaceous lesions of the face should prompt consideration of an underlying vascular etiology [3, 6]. Early recognition and appropriate imaging are essential to prevent disease progression and achieve optimal outcomes [1].

A key learning point from this case is the presence of clinicopathological discordance, which should prompt reconsideration of the initial diagnosis. Similar atypical presentations of arteriovenous malformations have been reported in the literature, where lesions lacking classical vascular features may lead to misdiagnosis as benign dermatological conditions [3, 5]. This highlights the limitation of relying solely on superficial biopsy in evaluating persistent facial lesions, as deeper vascular components may be missed [7].

The differential diagnosis in such cases includes sebaceous gland hyperplasia, rosacea, granulomatous dermatoses, and adnexal tumors. However, progressive enlargement, lack of response to conventional therapy, and a prolonged clinical course should raise suspicion of an underlying vascular anomaly [4, 6].

From a clinical perspective, early use of Doppler ultrasonography is recommended in persistent or atypical lesions, especially when histopathological findings do not correlate with clinical behavior. Further confirmation with contrast‐enhanced imaging is essential for accurate diagnosis and management [8, 9].

## Author Contributions

**Qazi Syed Irfanullah Shah:** conceptualization, data curation, methodology, writing – original draft. **Xuefeng Wan:** supervision, writing – review and editing. **Palida Abliz:** project administration, supervision, writing – original draft, writing – review and editing. **Akebaier Sulaiman:** conceptualization, data curation, investigation.

## Funding

This work was supported by the research grant “Research on the Microenvironment of Malignant Melanoma” (Project number: TSYC202301B058).

## Ethics Statement

Written informed consent was obtained from the patient for publication of this case report and all accompanying images.

## Conflicts of Interest

The authors declare no conflicts of interest.

## Data Availability

The data that support the findings of this study are available from the corresponding author upon reasonable request. The data are not publicly available due to privacy and ethical restrictions related to patient confidentiality.
